# “Every Newborn-INDEPTH” (EN-INDEPTH) study protocol for a randomised comparison of household survey modules for measuring stillbirths and neonatal deaths in five Health and Demographic Surveillance sites

**DOI:** 10.7189/jogh.09.010901

**Published:** 2019-06

**Authors:** Angela Baschieri, Vladimir S Gordeev, Joseph Akuze, Doris Kwesiga, Hannah Blencowe, Simon Cousens, Peter Waiswa, Ane B Fisker, Sanne M Thysen, Amabelia Rodrigues, Gashaw A Biks, Solomon M Abebe, Kassahun A Gelaye, Mezgebu Y Mengistu, Bisrat M Geremew, Tadesse G Delele, Adane K Tesega, Temesgen A Yitayew, Simon Kasasa, Edward Galiwango, Davis Natukwatsa, Dan Kajungu, Yeetey AK Enuameh, Obed E Nettey, Francis Dzabeng, Seeba Amenga-Etego, Sam K Newton, Alexander A Manu, Charlotte Tawiah, Kwaku P Asante, Seth Owusu-Agyei, Nurul Alam, M M Haider, Sayed S Alam, Fred Arnold, Peter Byass, Trevor N Croft, Kobus Herbst, Sunita Kishor, Florina Serbanescu, Joy E Lawn

**Affiliations:** 1 Maternal, Adolescent, Reproductive and Child Health (MARCH) Centre, London School of Hygiene &Tropical Medicine, London, United Kingdom; 2 School of Public Health, Makerere University, Kampala, Uganda; 3 INDEPTH Network Maternal, Newborn and Child Health Working Group Technical Secretariat; 4 Bandim Health Project, Bissau, Guinea-Bissau; 5 Research Center for Vitamins and Vaccines, Bandim Health Project, Statens Serum Institut, Copenhagen, Denmark; 6 OPEN, Odense Patient data Explorative Network, Odense University Hospital/Institute of Clinical Research, University of Southern Denmark, Odense, Denmark; 7 Center for Global Health, Department of Public Health, Aarhus University Denmark, Aarhus, Denmark; 8 Institute of Public Health, College of Medicine and Health Sciences, University of Gondar, Gondar, Ethiopia; 9 IgangaMayuge HDSS, Uganda; 10 Kwame Nkrumah University of Science and Technology, Kumasi, Ashanti, Ghana; 11 Kintampo Health Research Centre, Kintampo, Ghana; 12 University of Health and Allied Sciences, Kintampo Health Research Centre, Kintampo, Ghana; 13 Malaria Centre, London School of Hygiene &Tropical Medicine, London, United Kingdom; 14 Health Systems and Population Studies Division, icddr,b, Dhaka, Bangladesh; 15 ICF, Rockville, Maryland, USA; 16 Department of Epidemiology & Global Health, Umeå University, Umeå, Sweden; 17 Africa Health Research Institute, South Africa; 18 The DHS Program, ICF Rockville, Maryland, USA; 19 Centers for Disease Control and Prevention, Division of reproductive Health, USA

## Abstract

**Background:**

Under-five and maternal mortality were halved in the Millennium Development Goals (MDG) era, with slower reductions for 2.6 million neonatal deaths and 2.6 million stillbirths. The Every Newborn Action Plan aims to accelerate progress towards national targets, and includes an ambitious Measurement Improvement Roadmap. Population-based household surveys, notably Demographic and Health Surveys (DHS) and Multiple Indicator Cluster Surveys, are major sources of population-level data on child mortality in countries with weaker civil registration and vital statistics systems, where over two-thirds of global child deaths occur. To estimate neonatal/child mortality and pregnancy outcomes (stillbirths, miscarriages, birthweight, gestational age) the most common direct methods are: (1) the standard DHS-7 with Full Birth History with additional questions on pregnancy losses in the past 5 years (FBH^+^) or (2) a Full Pregnancy History (FPH). No direct comparison of these two methods has been undertaken, although descriptive analyses suggest that the FBH^+^ may underestimate mortality rates particularly for stillbirths.

**Methods:**

This is the protocol paper for the Every Newborn-INDEPTH study (INDEPTH Network, International Network for the Demographic Evaluation of Populations and their Health Every Newborn, Every Newborn Action Plan), aiming to undertake a randomised comparison of FBH^+^ and FPH to measure pregnancy outcomes in a household survey in five selected INDEPTH Network sites in Africa and South Asia (Bandim in urban and rural Guinea-Bissau; Dabat in Ethiopia; IgangaMayuge in Uganda; Kintampo in Ghana; Matlab in Bangladesh). The survey will reach >68 000 pregnancies to assess if there is ≥15% difference in stillbirth rates. Additional questions will capture birthweight, gestational age, birth/death certification, termination of pregnancy and fertility intentions. The World Bank’s Survey Solutions platform will be tailored for data collection, including recording paradata to evaluate timing. A mixed methods assessment of barriers and enablers to reporting of pregnancy and adverse pregnancy outcomes will be undertaken.

**Conclusions:**

This large-scale study is the first randomised comparison of these two methods to capture pregnancy outcomes. Results are expected to inform the evidence base for survey methodology, especially in DHS, regarding capture of stillbirths and other outcomes, notably neonatal deaths, abortions (spontaneous and induced), birthweight and gestational age. In addition, this study will inform strategies to improve health and demographic surveillance capture of neonatal/child mortality and pregnancy outcomes.

Almost nine million women and children die each year, two-thirds during pregnancy and around the time of birth [1]. An estimated 2.6 million babies are stillborn (die in the last three months of pregnancy or during childbirth) [2], 2.6 million liveborn babies die within the first 28 days of life (neonatal deaths) [1], and 303 000 women die of pregnancy complications per year [3]. Whilst child and maternal mortality rates halved during the Millennium Development Goal era, slower progress has been made for preventing stillbirths and neonatal deaths [4]. To accelerate progress, the Every Newborn Action Plan (Every Newborn) was launched in June 2014 [5], including national targets of 12 or fewer neonatal deaths per 1000 live births and 12 or fewer stillbirths per 1000 total births by 2030 [5,6]. Neonatal mortality is also a sub-target under the third Sustainable Development Goal (SDG 3). Both neonatal and stillbirth rates (SBR) are tracked in the United Nation’s Global Strategy for Women’s, Children’s and Adolescent’s Health 2016-2030 [6,7]. The countries needing the greatest acceleration to meet these targets are mainly in sub-Saharan Africa and South Asia, with both the highest risk of mortality and the lowest availability of data. To track SDG progress and inform investments towards the Every Newborn targets, data are essential on both coverage of interventions and impact.

In response, the World Health Organization (WHO) and the London School of Hygiene & Tropical Medicine (LSHTM) published an ambitious Every Newborn Measurement Improvement Roadmap. This Roadmap prioritises specific measurement gaps and provides a multi-year, multi-partner pathway to test validity of selected coverage indicators [8], develop tools (eg, improved birth and death registration, audit, minimum perinatal data set, gestational age and birthweight), and promote use of data by 2020 [5,9,10]. The roadmap includes improved measurement and classification of pregnancy outcomes including stillbirths, miscarriage, or termination of pregnancy (TOP) and neonatal deaths. Data on birthweight, gestational age and vital status at birth are critical for correct classification of these outcomes (**Figure 1**) [12].

**Figure 1:**
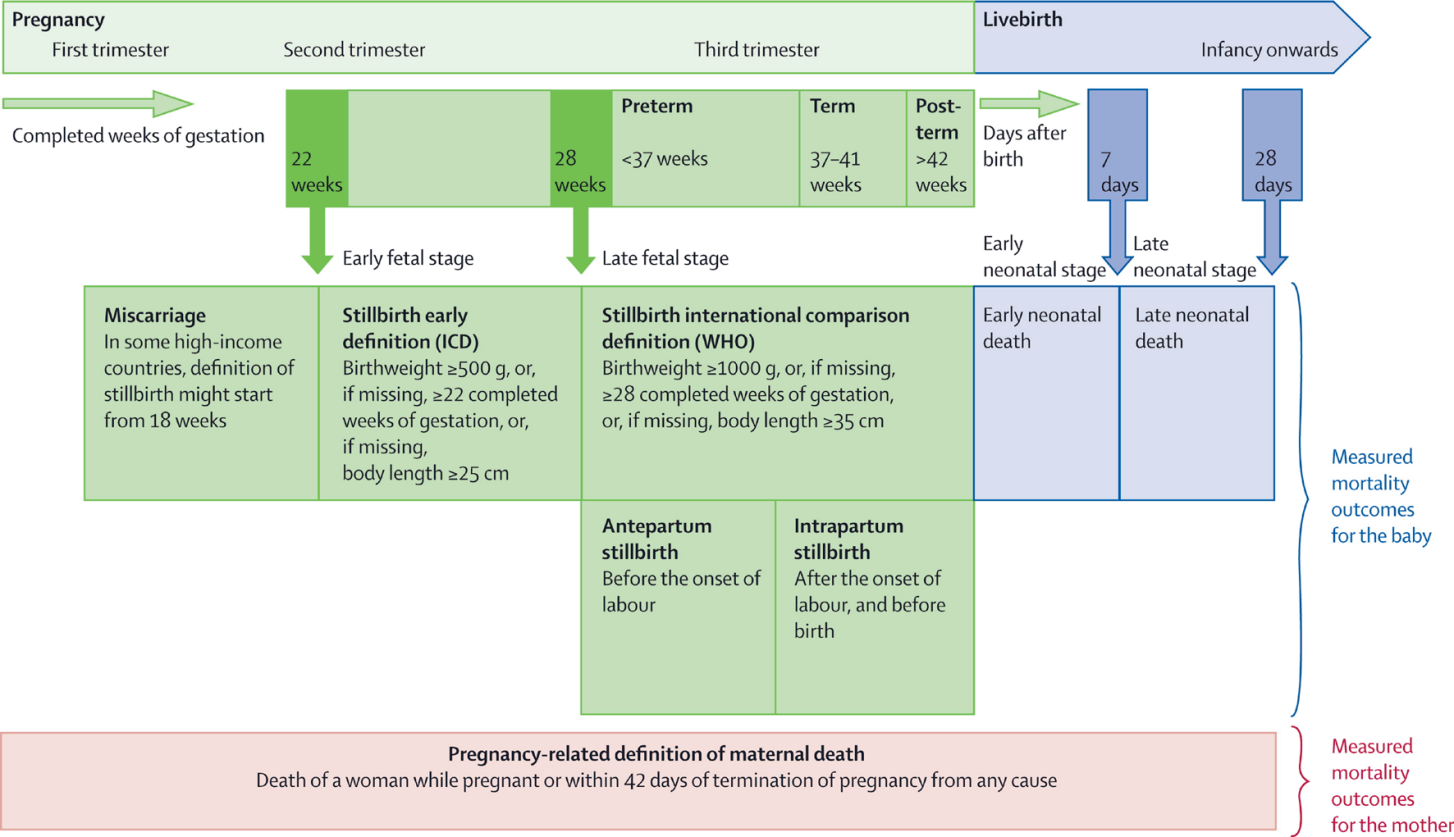
Pregnancy outcomes and neonatal deaths with definitions for international comparison. From [11]. Pregnancy outcomes include miscarriage, stillbirth, termination of pregnancy, gestational age at birth and birthweight. This figure does not include induced termination of pregnancy which are defined as an induced termination of pregnancy by medical or surgical means and this definition be different in countries depending on their law and regulations.

Population-based household surveys are the major source of population-level data on child and neonatal mortality rates (NMR) in settings without high coverage Civil Registration and Vital Statistics (CRVS) systems, and will continue to be an important source of data in the SDG-era [13,14]. Surveys are also sources for adverse pregnancy outcome data including stillbirths, miscarriages, TOPs, and birthweight, and gestation age.

Birthweight data are collected in surveys from either health cards or maternal recall. Where this information is not available, surveys ask about ‘maternal perceived size at birth’, which has been used in the estimation of low birthweight rates from surveys [15]. Surveys have a high proportion of missing birthweight data and heaping of reported birthweights [15-17]. Gestational age data are not usually presented in the survey reports, although these data are collected in months in the reproductive calendar for live births and stillbirths, and in additional questions for non-live births (miscarriages and abortions), eg, “How many months pregnant were you when that pregnancy ended?”. This answer relies on mother’s recall of the length of her pregnancy. The usefulness and validity of these survey data on gestational age in months are not known. More information on gestational age is now increasingly available from health facilities, where the last menstrual period can be recorded by a clinician and may be supplemented with an early ultrasound scan, fundal height during pregnancy, or clinical assessment of the newborn. Birth registration coverage is also assessed through household surveys, and these questions have not been assessed for feasibility or acceptability.

Although DHS collects data on abortions, most of these questions do not distinguish between induced abortions (TOP) or spontaneous abortions (miscarriages) and early/late fetal deaths or stillbirths [18]. This may contribute to undercounting of these different pregnancy outcomes. In some countries like Armenia and Nepal, the reproductive and health surveys go into more detail in collecting data on abortions, including the number of abortions and reasons why. A challenge with accurate capture of induced abortions is a reluctance to report, especially where they are illegal.

Currently, DHS use two alternative approaches to estimate NMR and SBR (**Figure 2**, panel A and panel B):

**Figure 2:**
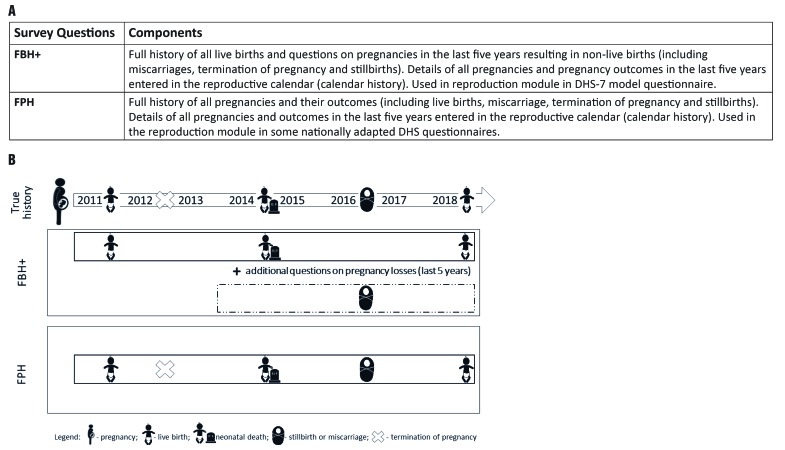
Two DHS alternative approaches to estimate neonatal mortality rates and stillbirth rates. **Panel A.** Full Birth History (FBH^+^) and Full Pregnancy History (FPH) approaches used to collect pregnancy outcomes including stillbirth and neonatal death. DHS-6 and DHS-5 also collected similar information with a Full Birth History, but information on pregnancies not resulting in a live birth were collected in the reproductive calendar only. The new design of DHS-7 questionnaire has additional questions inserted in the questionnaire after the Full Birth History to capture this information. **Panel B.** Data capture by FBH^+^ and FPH methodologies.

1. ***Standard DHS-7 core questionnaire Full Birth History (FBH^+^)***, with additional questions on pregnancies in the last five years resulting in a non-live birth used by a large majority of countries running DHS surveys;
2. ***Full Pregnancy History (FPH)***, used only in some countries in Eastern Europe and Central Asia, and more recently Afghanistan, Ghana, Nepal, the Philippines, South Africa, Vietnam.

The main difference between these two is that in FPH data are collected on all pregnancies in a woman's lifetime; whereas in the FBH^+^ data are collected on all live births in a woman’s lifetime and on any pregnancies not resulting in a live birth only for the five years preceding the survey.

Few comparisons of these two methods are available and no rigorous evaluation has been undertaken. No studies have directly compared the performance of the two methods in estimating SBR, but an indirect comparison between two contemporaneous surveys in Ghana: (1) 2008 DHS (using the standard FBH^+^) and (2) 2007 Maternal Health Survey (using FPH) found SBR were 35% lower using the FBH^+^ but there was no difference in Early Neonatal Mortality Rates (ENMR) [19,20] (Appendix S1 in **Online Supplementary Document**). Another study in the Philippines compared two surveys, both using a FPH, found 34% higher SBR when the FPH was part of a short maternal health survey (SBR 12.5 per 1000 total births), compared to when it was administered as part of a full DHS (SBR 9.6 per 1000 total births) [21]. The difference observed may have been due to a shorter, more focused questionnaire in the maternal health survey and not by the question structure itself. Only one published study has made a direct (non-randomised) comparison between the two methods for ENMR, and found 2%-3% higher rates using the FPH [22].

Whilst it is plausible that a FPH may yield improved capture of adverse pregnancy outcomes, it may be more time consuming and hence, data are required to show evidence of better capture before recommending this as standard in the already long DHS core questionnaire. In addition, it is important to understand interview procedures and barriers and enablers to reporting pregnancies and adverse outcomes, which have been understudied. Data from Tanzania suggests that socio-economic and cultural factors affect the quality of information collected on adverse pregnancy outcomes [23]. An analysis of 39 DHS suggested that fieldwork procedures affect data quality, notably including sex of the interviewer; whether or not a translator was used; the timing of the interview; and how many call-backs an interviewer had to make [24]. Some studies have reported on the stigma for women after stillbirths, newborn deaths and abortions in developing country contexts [25-27].

## Aim

This is the protocol paper for the **E**very **N**ewborn-**INDEPTH** Network (EN-INDEPTH) study which is part of the Every Newborn Measurement Improvement Roadmap, and aims to improve household survey capture of stillbirths and neonatal deaths by assessing whether FPH leads to increased capture of selected pregnancy outcomes compared to the standard DHS-7 FBH^+^ (**Figure 2**, panel A and panel B). The study will investigate the performance of existing or modified survey questions regarding other important measures related to pregnancy related outcomes, including fertility intentions, TOP, birthweight, gestational age, and coverage of birth and death certification. In addition, the study will examine barriers and enablers to reporting of pregnancy and adverse pregnancy outcomes in surveys and through Health and Demographic Surveillance Systems (HDSS).

### Research objectives

Research objectives, research questions and data analysis methods are summarised in **Table 1****.**

**Table 1:** EN-INDEPTH study summary of research questions and data analysis approach, according to the four study objectives.

Research objective	Research question	Data analysis approach
**Objective 1: Full Birth History (FBH^+^) approach vs Full Pregnancy History (FPH) approach**		
To undertake a randomised comparison of the reproductive module used in the latest version of FBH^+^ vs a FPH module to examine the variation in capture of stillbirths and neonatal deaths.	Is the FPH method better at capturing stillbirths and neonatal deaths in the last five years than the FBH^+^?	Descriptive and bivariate analyses comparing the two methods including meta-analysis: SBR; and NMR.
	How long does it take to collect data using the FPH questionnaire? Does the length of data collection vary by context and/or fertility level?	Bivariate analyses of the FPH and FBH^+^ by the time spent answering the questionnaires, variation by context and maternal characteristics.
**Objective 2: Pregnancy outcomes**		
To evaluate the use of existing/modified survey questions to capture the fertility intentions and selected pregnancy outcomes (top, miscarriage, birthweight, gestational age), as well as birth and death certification.	What is the answerability and data quality by indicator?	Descriptive analyses of selected indicators, and assessment of data quality per indicator (eg, non-response, heaping, missingness).
	How long does it take to collect data regarding these indicators? Does the length of data collection vary by data collector context and/or fertility level?	Analyses of survey paradata to assess variation by data collector (eg, gender, education level and training), time of day, rural/urban location, and time needed to complete survey questions and sections, frequency of repeated corrections of answers to questions.
**Objective 3: Survey vs HDSS data collection platforms**		
To compare the capture of pregnancy outcomes in the survey to that in the routine HDSS data collection	How do outcomes reported in the EN-INDEPTH survey compare with HDSS data?	Assess level of agreement at population-level between survey and routine HDSS data over the same time period for several indicators: SBR, NMR, miscarriage, TOP, birthweight, GA.
		For individually linked data, compare capture of pregnancy outcomes between survey and HDSS and assess predictors of capture.
**Objective 4: Barriers and enablers to reporting (adverse) pregnancy outcomes**		
To identify barriers and enablers to the reporting of pregnancy and adverse pregnancy outcomes during the survey and HDSS data collection, and particularly if these differ for the two survey questionnaire methods (FBH^+^ and FPH).	What are barriers and enablers to reporting of pregnancies and pregnancy outcomes (geographic, socioeconomic, cultural, data collection methodologies, etc.) in HDSS and survey data collection?	Quantitative analyses.
		Qualitative analyses of FGDs or IDIs for:
	What are interviewers’ perceptions (both HDSS and survey interviewers) of barriers and enablers to collect data on pregnancy losses in survey setting?	- survey interviewers
		- HDSS interviewers
		- supervisors
		- mothers who had a pregnancy in the past five years
	What are women’s perceptions and barriers for reporting pregnancy losses?	A priori coding. Use of the grounded theory and identify emerging themes and outliers; relationships and theories.
	How can data collection process be improved to obtain better data on adverse pregnancy outcomes?	

FBH^+^ – Full Birth History^+^; FPH – Full Pregnancy History; SBR – stillbirth rates ; NMR – neonatal mortality rates; TOP – termination of pregnancy; HDSS – Health and Demographic Surveillance Systems; GA – gestation age; FGDs – focus group discussions ; IDIs – in-depth interviews

**Objective 1. FBH^+^ vs FPH approach:** To undertake a randomised comparison of the reproductive module used in the latest version of FBH^+^ vs a FPH module to examine the variation in capture of stillbirths and neonatal deaths.

**Objective 2**. **Pregnancy outcomes**: To evaluate the use of existing/modified survey questions to capture the fertility intentions and selected pregnancy outcomes (TOP, miscarriage, birthweight, gestational age), as well as birth and death certification.

**Objective 3. Survey vs HDSS data collection platforms:** To compare the capture of pregnancy outcomes in the survey to that in the routine HDSS data collection.

**Objective 4. Barriers and enablers to reporting (adverse) pregnancy outcomes**: To identify barriers and enablers to the reporting of pregnancy and adverse pregnancy outcomes during the survey and HDSS data collection, and particularly if these differ for the two survey questionnaire methods (FBH^+^ and FPH).

## METHODS

### Study design

This multi-site study will use a retrospective survey to compare two methods of recording pregnancy outcomes (FBH^+^ vs FPH methods), with random allocation at the individual woman level. Quantitative and qualitative data will be collected to answer four research objectives (**Table 1**).

Research protocol development was informed by wide consultation, including review by the research site teams and an Expert Advisory Group. 23 participants took part in a study design workshop in Kampala in mid-2016 [28]. In addition, in April 2017, a multi-site workshop was organised to agree on the data collection protocol in the five sites.

### Study settings

The INDEPTH Network’s HDSS is a network of research sites in 53 countries, established in 1998. Each site tracks vital events in a defined population on a continuous basis, but methods used vary [29]. Since some sites undertake pregnancy surveillance, the INDEPTH network provides an ideal platform for this multi-site retrospective population-based survey with a potential for linking with prospective HDSS data. The INDEPTH Network operates through Working Groups, one of which, the Maternal Newborn and Child Health (MNCH) working group [30], hosted by the Makerere University School of Public Health (MakSPH), Uganda, will coordinate this study, partnering with LSHTM.

**Figure 3:**
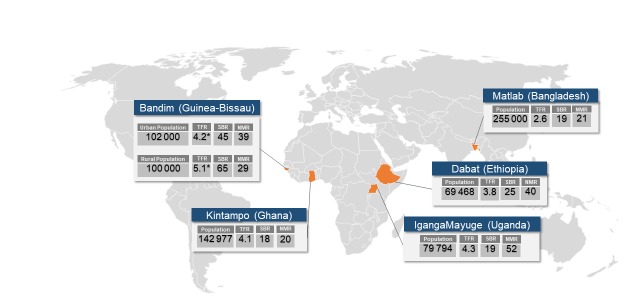
Map showing the location of the EN-INDEPTH study HDSS sites. Total fertility rate (TFR) for women ages 15-49; neonatal mortality rate (NMR) per 1000 live births; stillbirth rate (SBR) per 1000 live births. More detailed information on study HDSS sites: Bandim (http://www.indepth-network.org/member-centres/bandim-hdss); Dabat (http://www.indepth-network.org/member-centres/dabat-hdss); Iganga-Mayuge (http://www.indepth-network.org/member-centres/igangamayuge); Kintampo (http://www.indepth-network.org/member-centres/kintampo-hdss); Matlab (http://www.indepth-network.org/member-centres/matlab-hdss). Asterisk: Bandim – children/pregnancies only followed prospectively; TFR estimated by cumulative birth hazards (Nelson Ahlen) as observed for specific age bands between 2012-16 extrapolated to age span 15-50 years; SBR, NMR estimated among registered pregnancies ending in 2012-16.

A Request For Applications (RFA) was sent to all 53 HDSS sites in December 2015 by the INDEPTH Network secretariat. Fourteen proposals were received and reviewed by LSHTM, MakSPH and the INDEPTH Network secretariat [31]. The selection criteria were: HDSS total population of more than 30 000 people; annual SBR and NMR greater than 15 per 1000 total births; acceptable quality surveillance for pregnancy outcomes, including neonatal deaths and stillbirths; expertise on maternal and newborn health, and stillbirths among the HDSS team members and evidence of co-funding in the submitted estimated budgets. Five sites (Bandim, Dabat, IgangaMayuge, Kintampo, and Matlab) were selected (**Table 2** and **Figure 3**), all of which, as well as LSHTM, received ethical approval from local institutional review boards (Appendix S2 in **Online Sup**plement**ary Document**).

**Table 2:** Expected sample size across the five INDEPTH sites

Characteristics	Bandim	Matlab	Kintampo	Dabat	Igangamayuge	Across the five sites
**Estimated number of total births over the five years captured within the HDSS**	29 173	25 799	24 008	7031	11 489	97 499
**Surveillance system**	Bi-annual update rounds in the rural area and monthly updates in urban area. Update rounds includes registration of pregnancies	Two monthly update rounds including pregnancy testing and registration	Bi-annual update, from 2017 they have shifted to an annual update rounds	Bi-annual update rounds. Monthly updates of births and deaths from local guides.	Bi-annual update rounds. Monthly updates of births and deaths from local scouts.	
Sampling frame*	Women in HDSS site with recorded birth outcome in last 5 y. (all in urban site and 80% in rural site)	Women in HDSS site with recorded birth outcome in last 5 y. (all)	Women in HDSS site with recorded birth outcome in last 5 y. (random sample)	Women of reproductive age in HDSS site	Women of reproductive age in HDSS site	
**Expected number of total births to be captured in survey**	17 000	21 000	14 500	5700	9800	68 000

HDSS – Health and Demographic Surveillance Systems

*See Appendix S3 in **Online Supplementary Document** for details.

While all the selected HDSS sites undertake pregnancy surveillance, the quality of which is influenced by various factors including: frequency of surveillance rounds; the key informants ratio to population; gender; the method used to ask about pregnancy and pregnancy outcomes; the proportion of facility births; and linkage between HDSS and facility data. Cultural norms around pregnancy disclosure may also vary and affect the data collected (**Table 2**).

### Sample size and data collection approach

Limited evidence from previous descriptive studies discussed above suggests a difference in SBR from 3 to 35% between a FBH^+^ and a FPH method [19,20]. A difference of at least 15% between the two methods could be sufficient to consider a major change in the DHS core questionnaire. Since this is the first direct comparisons of the two methodologies, we powered the randomized comparison to capture a 15% difference assuming that a difference less than 15% will not justify a change in survey methodology (see Appendix S1 in **Online Supplementary Document**).

Based on the recorded SBR and total births per HDSS over the last three years, the overall SBR for the final sample of births captured in the survey is expected to be around 28.4 per 1000 total births, if all women are surveyed. However, we needed to account for possible outmigration, unavailability of women at any visit, or non-consent. After adjusting for these factors, we expect the SBR for the FPH arm to be around 26.7 per 1000 total births (see Appendix S3, Table S2.2 in **Online Supplementary Document** for details). Assuming a SBR of 26.7 per 1000 total births in the FPH arm, a total sample size of at least 68 000 births would be required across the five sites to have 80% power to detect a difference of 15% or more between the proportion of total births that are stillbirths in the FBH^+^ and the FPH at the 5% significance level (alpha = 0.05), including a small design effect (DEFF = 1.1), as stillbirths may be clustered in individual women. The lower the SBR captured in the FBH^+^ arm, the higher the sample size will be required to detect 15% difference between the two arms (see **Table 3**).

**Table 3:** Required sample size by stillbirth rate (SBR) for the household survey randomised comparison, assuming alpha = 0.05 and an expected 15% difference in SBR*

Assumed SBR in birth history group/ 1000 total births	Predicted SBR in pregnancy history group/ 1000 total births	Number of total births to achieve 80% power	Sample size - number of births required including design effect and non-response (15%)
23.00	26.45	63 604	80 459
23.20	**26.70**	**62 348**	**68 583**
24.00	27.60	60 886	77 021
25.00	28.75	58 386	73 858
26.00	29.90	56 078	70 939
27.00	31.05	53 942	68 237
28.00	32.20	51 958	65 727

*The Design effect (DEFF) is calculated as DEFF = 1 + (r – 1) × rho, where r = average number of observations in a cluster and rho = correlation between pairs of observations selected at random from the same cluster). Assuming in a 5-year period women will experience on average a maximum of 2 births, and that as stillbirth is a rare outcome rho<0.1, a design effect of 1.1 is included. For Bandim, due to the challenge of reaching women in rural area, we can only account for a maximum of two visits to reach the interview, for this reason we have assumed a higher rate of non-response rate (30%).

For each site the approach to reach the maximum number of births was agreed based on site-specific information on the level of migration, geographical accessibility, and assumed levels of non-response (either due to non-consent or inability to locate) and other factors. The numbers per site are shown in **Table 2**and details of the estimation are in Appendix S3, Table S2.2 in **Online Supplementary Document**. Three sites (ie, Matlab, Dabat and IgangaMayuge) will survey all women who have given birth in the last five years. Matlab has bi-monthly data collection with pregnancy testing for women who report having missed a menstrual period, and hence has reliable listings of total births, and will administer the EN-INDEPTH survey to eligible women (age 15-49) known to have had at least one birth (live birth or stillbirth) in the last five years; Dabat and IgangaMayuge have bi-annual updates and do not have a specific system of pregnancy tracking and therefore, all women of reproductive age (15-49 years) in the HDSS site will be included. Bandim and Kintampo, will select a random sample of women residing in the HDSS known to have at least one birth in the last five years (**Table 2**).

### Data collection software application

Currently, the DHS and Multiple Indicator Cluster Survey (MICS) surveys are administered either using a paper version of the questionnaire (PAPI) or computer assisted personal interviewing (CAPI) and the CSPro (Census and Survey Processing System) platform for entering the data entry or data capture. We elected to use Android-based tablet data collection as most of the selected HDSS sites had some experience with these. We compared several existing data collection platforms (ODK, CSPro, Qualtrics, Red Hat and Survey Solutions). We selected the Survey Solutions Computer Assisted Personal Interview and Computer Assisted Web Interviewing platform (Survey Solutions) developed by the World Bank [32], given its ability of linking questions to specific household members listed in the roster file; its flexible and user-friendly online interface for the questionnaire design; the ability to integrate validation rules; lack of user fees; the online tool for centralised survey administration and data management; data aggregation and reporting features; end-user technical support provided by the World Bank technical support team; as well as the Interviewer application available for Android devices.

**Figure 4:**
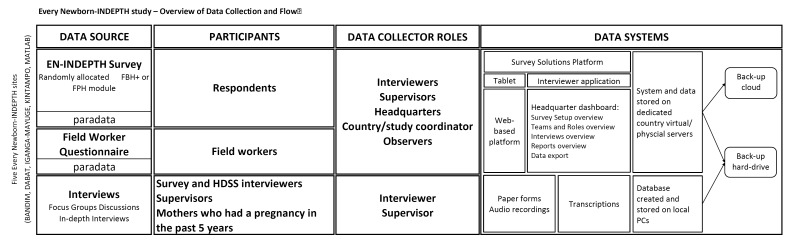
EN-INDEPTH Data Collection and Flow.

The data will be entered on Android-based tablets using the Survey Solution platform, stored locally on the tablets, and synchronised regularly to the dedicated country’s physical or virtual server. The platform has different user roles with varying level of permissions and functions: Interviewer (function – data collection), Supervisor (assigning and monitoring data collection by interviewers), Headquarters (overall survey and data management), and Observer (monitoring) (**Figure 4**). The platform’s survey and data management component (‘Survey Solutions Headquarters’, HQ) provides a dashboard of the progress of data collection, including duration and speed of the interviews, Global Positioning System coordinates of the interviewer, as well as paradata. The paradata contains information about the process of collecting survey data and records all events with timestamps on a tablet during data collection (data entry, data correction, responsibility changes, etc.). This type of data can be used for analysis of time per interview and time per question and section, as well as changes in productivity over time for different interviewers and teams. These data can be used for live data quality control, data monitoring and interview progress evaluation.

### Procedures

#### Informed consent and respondent identification

All participants will receive a verbal explanation of the study objective by a trained field assistant and an information sheet. An adapted version of the standard DHS consent form will be used (Appendix S4 in **Online Supplementary Document**). Both will be translated into local languages and written consent will be obtained from all study participants. Study participants will be informed of their right to refuse and/or withdraw at any point of time from the survey interviews, and at liberty to answer those questions they want to and will not be coerced in case they refuse. Information obtained will be viewed only by the researchers and anonymity will be guaranteed by using identification numbers. Separate written consent will be sought for women and interviewers participating in the qualitative aspects. We will follow fieldwork procedure as outlined in the DHS interviewer manual. Interviewers will make up to three attempts to find respondents.

### Randomisation and EN-INDEPTH survey

Women selected for the survey from all five sites will be randomised to receive either the reproduction questions from the DHS-7 women’s questionnaire with a standard FBH^+^ or a FPH. Randomisation will be done automatically within the application-based questionnaire using an in-built random number generator.

**Table 4** provides a summary of adaptations to the DHS-7 questionnaire sections to meet the study objectives including questions on gestational age, birth certification, characteristics of stillbirths and fertility intentions. The latest DHS questionnaire (DHS-7) will be used, with a shortened introduction section 1 of the DHS-7 women’s questionnaire; section 2 on reproduction; section 4 on pregnancy, delivery and postnatal care; and an additional section 9 adapted on household characteristics. Section 8 on fertility intentions was optional and the Matlab site opted not to administer this as they have just recently completed the collection of similar data. Some specific country add-on modifications will be made to the questionnaire (eg, Bandim site will collect questions on vaccinations, and the IgangaMayuge site will collect data using the Dietary Diversity questionnaire in section 10). Apart from section 2, the questionnaires for the FBH^+^ and FPH arms will be identical. Section 2 in the FBH^+^ arm will contain the standard DHS-7 core reproduction questions. In the FPH arm, section 2 will contain the whole reproduction section from the Nepal 2016 DHS for sites willing to include detailed questions on abortion, and a reduced version without the detailed abortion questions for other sites. Appendix S5 in **Online Supplementary Document** gives full details of the questionnaires.

**Table 4:** Current standard DHS Phase 7 questionnaire sections and adaptations for this study

Survey questionnaire with FBH^+^ or FPH detailing adaptations from standard DHS phase 7 section, where applicable	
**Section 1. Consent form and background of interview**	The content of section 1 will be reduced to focus on key maternal background characteristics only.
**Section 2. Reproduction**	Standard FBH^+^ questions with pregnancy loss questions which include information on stillbirths, miscarriages and abortions. No adaptations made. Or randomly allocated to FPH from Nepal 2016 for the FPH and pregnancy losses questions – the detailed questions on abortion will only be administered in selected sites.
**Section 4. Pregnancy and postnatal care**	Section 4 will be administered with minor adaptations for all stillbirths and neonatal deaths, as well as for a sample of live births. Additional questions on gestational age (weeks), birth and death certification, and timing and characteristics of stillbirths will be added to test the feasibility of these questions in household surveys.
**Section 8. Fertility preferences**	Some questions on fertility intention to refine the measurement on unwanted pregnancy will be added. These questions have been developed and tested in a multi-country research study [33].
**Section 9. Household characteristics**	Questions on household socio-economic characteristics including household dwelling structure, flooring material, sanitation and toilet facility. These questions are adapted from the DHS household survey questionnaire.

FBH^+^ – full birth history +; FPH – full pregnancy history; DHS – Demographic and Health Surveys.

Local language translations of the questions already used by DHS will be used whenever available. Where not available, translations to local languages will be made by the site teams and checked using back translation.

#### Assessment of barriers and enablers to reporting of pregnancy and adverse pregnancy outcomes

For objective 4, a self-administered questionnaire adapted from DHS fieldworker questionnaire, will assess demographic and other characteristics of the interviewers (Appendix S6 in **Online Supplementary Document**). A series of Focus Group Discussions (FGDs) will be held with HDSS and survey interviewers, as well as supervisors, to assess the barriers or enablers to collecting data on pregnancy and adverse pregnancy outcomes in the survey and HDSS. FGDs will be conducted with women who had at least one pregnancy in the past five years, focusing on perceptions, practices, barriers and enablers in the community. A minimum of six FGDs will be conducted in each site, each with approximately eight to ten participants. In-Depth Interviews (IDIs) will be undertaken in some sites with women who have experienced these adverse pregnancy outcomes, allowing for deeper exploration and triangulation of data.

#### Training of data collectors and supervisors

The EN-INDEPTH site teams with LSHTM and MakSPH jointly developed a training manual on the data collection procedures, adapting the standard DHS Interviewer’s Manual [34], and tailored it to the specific country context and the HDSS site. Four additional manuals (data collection setup, Survey Solutions data management procedures, listing process, Survey Solutions Tester/Interviewer application) were developed adapting the World Bank Survey Solutions Manuals [35]. The training of data collectors and supervisors was led by the HDSS team with initial support from the core team for a minimum of two weeks in all HDSS sites. The training included at least one-week on the paper-based questionnaire and one to two weeks on tablet use, data collection using the Survey Solutions Tester application, as well as the Survey Solutions Interviewer application and interviewer field practice. Prior to data collection, additional trainings on survey management using Survey Solutions HQ were provided to supervisors and data analysists.

For the qualitative work, training manuals will be developed to guide the interviewers during the FGDs and IDIs to ensure comparability of interviews across sites. Additionally, a protocol will be developed to guide the interviewers on how to react in situations where the respondent gets distressed. After the initial two to three weeks training, all sites will initiate the pilot phase of data collection. The length of the data collection will vary by site depending on the fieldwork schedule and allocated sample size ranging between six to 12 months.

#### Data quality monitoring

Validations for value ranges were defined and programmed into the tablet application to avert predictable human errors. The skipping rules were programmed and additional rules were set to perform consistency checks. Warning messages were programmed to prompt to correct the input when values are outside the defined range, and to provide guidance as per the DHS manual. Data quality will be monitored using Survey Solutions platform’s online data dashboard, providing real-time cumulative and detailed summary of ongoing surveys across teams and individual interviewers in each country. The platform allows Supervisors and Headquarters to validate collected data by Interviewer online and, if necessary, incomplete or erroneous questionnaires can be returned to the Interviewer for timely re-assignment and correction. In addition, bi-weekly reports will be sent to the LSHTM and MakSPH core teams by data analysts from all sites summarising the overall data collection progress. Regular all-site data monitoring calls will provide an opportunity for country teams to review and discuss progress in addition to promoting collaborative quality improvement initiatives between countries and sites.

#### Data management

Following synchronisation, data from tablets will be uploaded to the country’s dedicated virtual or physical server with regular automatic back-up, with additional back-up on a separate server or external hard drive. The raw data will be stored in an encrypted format, accessed only by the country’s data manager. The anonymization of the quantitative and qualitative data (removing any direct or indirect identifiers, including enumeration identifiers, geo-referenced data, transcripts and audio recording) will take place in-country before data sets are pooled into one multi-site data set (**Figure 4**).

### Analysis by objective

The overview of research objectives, main research questions, and analytical approaches are summarised in **Table 1**. For all study objectives, the primarily analyses will be performed overall across countries (as pooled analyses), and comparative secondary analyses will be performed by site separately, whenever possible. Data will be cleaned according to an agreed protocol, including logical and completeness checks. Quantitative analyses will be undertaken with Stata 15SE (Stata Statistical Software: Release 15. College Station, TX: StataCorp LLC). Qualitative analyses will be conducted using NVivo software (NVivo qualitative data analysis Software; QSR International Pty Ltd Version 12, 2018).

#### Objective 1

A population-level descriptive analysis will be conducted comparing SBR and NMR by FBH^+^ or FPH, and by maternal characteristics (age, parity, residence, and education status). Crude risk ratios with its 95% confidence interval will be computed for comparison of SBR and NMR between FBH^+^ and FPH overall and by study site using the meta-analysis methods with Random Effects. Regression models will be fitted to assess determinants of adverse pregnancy outcomes using Generalised Estimation Equations to adjust for clustering of stillbirths or neonatal deaths within the same woman, therefore taking into account design effect. We will use paradata to assess differences in average time taken to complete the FBH^+^ and FPH.

#### Objective 2

We will undertake descriptive analyses of selected indicators including fertility intentions, selected pregnancy outcomes (TOP, miscarriage, birthweight, gestational age), as well as birth and death registration. This will include estimates of frequency of reported TOP and miscarriage, coverage of reported birthweight, gestational age, birth and death certification, and of fertility intentions. The answerability of new/refined questions will be assessed by describing patterns of non-response and heaping, where appropriate. Variation in these indicators by pregnancy outcome, maternal or interviewer characteristics will be assessed. For gestational age, internal consistency in the survey will be assessed by comparing women’s reporting of gestation in months compared to weeks, and reported maternal perception of the birth to be preterm. Survey paradata will also be analysed to assess time taken to complete and frequency of repeated corrections to relevant survey questions.

#### Objective 3

Women-level data from the survey will be individually matched with the routine HDSS surveillance data to establish matching rates for stillbirths and neonatal deaths. We will assess determinants of reporting or not reporting of these outcomes in the survey by women’s and interviewers’ characteristics and HDSS settings (geographic, socioeconomic, cultural, data collection methodologies, etc.). We will also assess levels of agreement between the survey and the routine HDSS data over the same time period at a population level for several indicators, such as SBR, NMR, miscarriage, TOP, birthweight, and gestational age. Predictors of disagreement (eg, length of recall, maternal education, etc.) will be examined. We recognize that neither HDSS nor survey data can be considered ‘gold standard’ and that the difference in measurement might be in both or either direction and variable by site.

#### Objective 4

For qualitative data, we will use a grounded theory approach for analysis, with an iterative process involving reading the text, detecting potential emerging themes and outliers, comparing themes and searching for relationships, as well as building theoretical models. A priori coding will be done, with a codebook listing potential codes developed before the analysis begins, to guide the process, and new codes identified from data included as analysis proceeds. Results will be presented with verbatim quotes from respondents. Reliability will be checked by multiple members of the team, two from each site, independently coding data.

## DISCUSSION

The EN-INDEPTH is the first randomised comparison of two survey methods to capture pregnancy outcomes, the current DHS-7 FBH^+^ and FPH. This is a large-scale study (at least 68 000 births) based in five high-burden countries, including one site in South Asia and four sites across West and East Africa. The study is powered to be able to detect a 15% difference in the estimated SBR, but it is also expected to inform our understanding of survey capture of other pregnancy outcomes, notably neonatal deaths, birthweight, gestational age and abortions (spontaneous and induced). Even if the results show a convincing increase in capture of stillbirths or other pregnancy outcomes, a key operational question is whether the FPH takes longer. The software used for our study (Survey Solutions) allows recording of the paradata, including precise timing by section of the questionnaire. This study will, therefore, enable us to conduct more detailed analyses of time spent by question and section, as well as by the fertility context. Underreporting of pregnancy and adverse outcomes may be affected by socio-cultural barriers and survey data collection procedures, so the mixed methods assessment of barriers and enablers to reporting and recording will be valuable.

In addition to omission of events, household surveys are known to have important limitations in the measurement of stillbirths and neonatal deaths, including displacement of reported day of death and misclassification between stillbirths and neonatal deaths [36,37]. Distinguishing between stillbirths and neonatal deaths requires detection of signs of life at birth, notably assessment of heart rate. Recall by a mother in a survey requires her to know whether there were signs of life at birth and for her to report this. Differences in assessment at birth, perceptions of viability, availability of neonatal resuscitation and cultural and religious factors - all potentially have a role in whether a mother will report her baby’s death as a stillbirth or an early neonatal death. This study will examine any differences between reporting of these outcomes in the survey compared to HDSS data, and explore women’s perceptions of stillbirth and neonatal deaths, but will not have the ability to assess “true” stillbirths based on lack of accurate measures of heart rate at birth. Another important misclassification is between early fetal death and late fetal death/stillbirth with a threshold of 28 weeks, based on errors in gestational age measurement and reporting. Again, this study will not have “true” gestational age based on first trimester ultrasound.

Birthweight and gestational age measurements are important from individual, clinical and public health perspectives. From a clinical perspective, they are important to identify liveborn neonates at increased risk of mortality and morbidity, for example, those preterm (born at <37 completed weeks of gestation) or low birthweight (<2500g), to enable provision of extra care [12]. From a classification perspective, this information is critical to differentiate between miscarriages and stillbirths. Studies have shown that data on perceived size at birth recorded in surveys are not consistent with data recorded from health cards and that the quality of recalled birthweight data are variable [38]. In addition, little is known on community perceptions of the importance of birthweight and how this may influence reporting. This study seeks to provide further insights on how to obtain better birthweight data in surveys. Although a gestational age in months is collected in the five-year reproductive calendar in DHS surveys, concerns have been raised regarding the validity of these data. Whilst months are used to differentiate between miscarriages and stillbirths, they are currently not reported in most survey reports and are not used in the estimation of population-level measures, such as preterm birth. In this study, we will assess standard and modified questions that seek to capture gestational age, as well as the internal validity of the reporting of gestational age in months, in weeks, and reported maternal perception of the birth as preterm. In the Matlab site, where more accurate gestational age information is captured in the HDSS through early routine urinary pregnancy testing following a missed period, we will assess the validity of these questions by comparing the information captured in the EN-INDEPTH survey to the HDSS data. This will provide important new information on the feasibility of the use of these questions in household surveys. However, the frequent HDSS household visits might increase women’s awareness of pregnancy duration and improve the reporting of gestational age.

Comparable data on abortion are limited [39]. In countries where abortion is illegal, data are underreported for fear of prosecution or stigma [18]. In contexts where abortion is legal, data may also be problematic. This study will add to the literature by testing the feasibility to asking a small set of abortion questions.

Fertility intentions are subject to substantial variability over time [40]. As part of a multi-country research protocol developed by the “STEP-UP” consortium that the LSHTM is co-leading with the Population Council [33], a set of new questions were developed to improve the measurement of ambivalence. We will test these questions, and because the study is nested in the HDSS sites, we will be able to prospectively assess the link between un-intendedness and pregnancy outcomes. The information collected will support a rigorous assessment of reasons for unmet need for family planning, as well as to assess whether unwanted childbearing is linked to negative pregnancy outcomes.

Registering a child’s birth is a critical first step to protect the rights of every child, and non-registration might prevent the child from accessing health and education services. UNICEF estimates suggest that more than 230 million children under the age of 5 have not had their birth registered [41]. Household surveys represent the largest source of data on birth registration in low- and middle-income countries, [41]. Yet, registration and notification procedures vary, so more research into context specific survey questions is required [42]. In consultation with experts in the Child Protection team in UNICEF HQ, we selected birth registration questions to be evaluated in this study.

Whilst this study has strengths in terms of being randomised for the primary objective, as well as being multi-site and large-scale, pregnancies resulting in a stillbirth are less likely to be captured than those resulting in a live birth, especially if there is no frequent surveillance to capture new pregnancies and live births, even within relatively robust pregnancy surveillance systems. Hence, one limitation of this method is that women with only a stillbirth in the last five years and no live birth would not be surveyed, potentially underestimating the true SBR. In addition, the sites selected for this study have slightly different surveillance systems. Since we are collecting the data in the context of the demographic surveillance system, respondents might already be familiar with these survey questions and may be more likely than women in other settings to report pregnancy losses. If this is the case, we might have a higher reporting of events than in other settings. However, if respondents in the community are indeed more likely to report such events, this should affect women in the HDSS site equally and should not affect the randomised experiment.

The choice of Survey Solutions as our data collection tool might affect comparability with other studies using PAPI or CAPI using the CSPro Windows tablet interface. The main difference between these methods of data collection is that both PAPI and CAPI using the CSPro Windows tablet interface have a roster for the data collection of reproductive histories. In order to minimise such effect, we developed a summary of the reproductive history to mimic the reproductive history roster used in the original PAPI or CAPI using the CSPro Windows tablet application. In addition, another strength of this study is that our customised survey questionnaire developed with Survey Solutions Designer allows the inclusion of interviewer’s instructions and these are visible to the interview for each question as the interviewer progresses during the interviewer visit. This addition might improve data quality.

## CONCLUSIONS

Most of the 5.5 million deaths around the time of birth [1] occur in countries with the least data. Whilst improvement in CRVS and routine facility data systems is crucial, in the meantime the poorest countries rely on household surveys, and equity considerations should drive investment to improve the quality of data capture, especially for the large burden of pregnancy outcomes. We anticipate that the results of this study will inform improved tools and how these tools are applied and enable better measurement of the often-hidden outcomes associated with stigma and suffering of women in many countries. Better data alone will not change these outcomes, but counting and visibility is a crucial first step to change.

## Additional material

Online Supplementary Document
