# Supplementary material for: “Every Newborn-INDEPTH” (EN-INDEPTH) study protocol for a randomised comparison of household survey modules for measuring stillbirths and neonatal deaths in five Health and Demographic Surveillance sites
Source: J Glob Health. 2019 Feb 15;9(1):010901. doi: 10.7189/jogh.09.010901 (PMC6377797; doi:10.7189/jogh.09.010901)
Supplement: Online Supplementary Document [file jogh-09-010901-s001.zip › Appendix.docx]

**Online Supplementary Document**

“Every Newborn-INDEPTH” (EN-INDEPTH) study protocol for a randomised comparison of household survey modules for measuring pregnancy outcomes in five Health and Demographic Surveillance sites

*Angela Baschieri; Vladimir S Gordeev; Joseph Akuze; Doris Kwesiga; Hannah Blencowe; Simon Cousens; Peter Waiswa; Ane B Fisker; Sanne M Thysen; Amabelia Rodrigues; Gashaw A Biks; Solomon M Abebe; Kassahun A Gelaye; Mezgebu Y Mengistu; Bisrat M Geremew; Tadesse G Delele; Adane K Tesega; Temesgen A Yitayew; Simon Kasasa; Edward Galiwango; Davis Natukwatsa; Dan Kajungu; Yeetey AK Enuameh; Obed E Nettey; Francis Dzabeng; Seeba Amenga-Etego; Sam K Newton; Alexander A Manu; Charlotte Tawiah; Kwaku P Asante; Seth Owusu-Agyei; Nurul Alam; M M Haider; Sayed S Alam; Fred Arnold; Peter Byass; Trevor Croft; Kobus Herbst; Sunita Kishor; Florina Serbanescu; Joy E Lawn**

**-* principal contact for editorial correspondence

Submitted to J Glob Health 2017

**Supplementary material for “Every Newborn-INDEPTH” (EN-INDEPTH) study protocol for a randomised comparison of household survey modules for measuring pregnancy outcomes in five Health and Demographic Surveillance sites”**

**Table of Contents**

[**Appendix S1** – Summary of available data regarding use of modules for pregnancy history versus birth history 2](#_Toc511134122)

[**Appendix S2** – Ethical Approval of local Institutional Review Boards 3](#_Toc511134123)

[**Appendix S3** – Study population – planned sampling strategy and expected weighted mean stillbirth rate from final EN-INDEPTH survey 4](#_Toc511134124)

[**Appendix S4** Consent forms 6](#_Toc511134126)

[**Appendix S5** – EN-INDEPTH survey questionnaire (separate Excel file) 13](#_Toc511134127)

[**Appendix S6** – Fieldworkers questionnaire (separate Excel file) 14](#_Toc511134128)

# Appendix S1 – Summary of available data regarding use of modules for FPH versus FBH+

| *Source* | *Reference* | *Study site* | *Dates of Study* | *Comparator 1* | *Comparator 2* | *Number of observations* | *Rates in comparator 1* | *Rates in comparator 2* | *Differences* |
| --- | --- | --- | --- | --- | --- | --- | --- | --- | --- |
| DHS program | Ghana Statistical Agency [13] | Ghana (national) | 2008 DHS (birth history) and 2007 Maternal Health Survey (FPH) | Standard DHS with FBH+ | Maternal Health Survey (MHS) with FPH | DHS – 2,949  MHS – 6,960 | Stillbirths  40/2949  13.6 per 1000 | Stillbirths  146/6960  21.0 per 1000 | Rates were 35% lower in birth history compared to pregnancy history  RR=0.65 (0.46 – 0.93)  There was no difference in Early Neonatal Death (ENND) between the two surveys  RR=1.08 (0.82 – 1.42) |
| DHS program | Ghana Statistical Agency[14] |  |  | 107 surveys using birth history | 13 surveys using pregnancy history |  | Mean SBR: ENMR ratio = 0.75  Mean SBR=12.7 | Mean SBR: ENMR ratio = 0.9  Mean SBR=13.3 |  |
| Older studies which were not direct comparison of the birth/pregnancy history approach, or did not assess SBR difference | | | | | | | | | |
| Stanton C. | PhD JHU  [15] | Philippines, National | 1993 DHS and SMS surveys | Standard DHS with FPH replacing birth history | Safe Motherhood Survey (SMS) – pregnancy history plus sex of stillbirth, additional probe if pregnancy interval ≥4years | In SMS 6329 births in three years prior to survey |  |  | # stillbirths 34% higher in SMS  # early losses 26% higher in SMS  [note comparison not direct as different household, same pregnancy history module but DHS many more questions – findings may be more about survey fatigue than the module] |
| Espeut D., Becker S. | Journal of Health, Population and Nutrition[16] | Bangladesh, Matlab HDSS | 1994 Matlab Demographic survey | 50% questionnaire FBH+ forward (as per Bangladesh DHS) | 50% questionnaire FPH forward | Matlab DHS – 3225 households sampled, 3009 household interviews, 3480 women reproductive age, 3039 women interviews | ENND – 46/58 (79%) (HDSS baseline) captured in survey  Late Neonatal Death (LNND) – 22/26 captured | ENND –  63/77 (82%) (HDSS baseline) captured in survey | Did not report on stillbirths  For ENNDs – 2 – 3% higher capture in pregnancy history  [note also very small sample size] |

# Appendix S2 – EN-INDEPTH study Ethical Approval of local Institutional Review Boards

| HDSS site | Institutional Review Boards | Date | Number/Ref |
| --- | --- | --- | --- |
| Bandim | Comité Nacional de Ética na Saúde | 12 June 2017 | 072/CNES/INASA/2017 |
| Dabat | Institutional Review Board, University of Gondar | 19 April 2017 | VP/RCS/05/1074/2016 |
| Iganga-Mayuge | Mildmay Uganda Research Ethics Committee  Uganda National Council of Science and Technology | 26 June 2017  11 October 2017 | REC REF 0305-2017  SS 4244 |
| Kintampo | Kintampo Health Research Centre, Ghana Health service  Ghana Health Services Ethics Review Committee  Kintampo Health Research Centre Institutional Ethics Committee | 14 June 2017  26 July 2017  9 August 2017 | SRC/130617  GHS-ERC:19/06/14  KHRCIEC/2017-14 |
| Matlab | Icddr,b Ethical Review Committee | 19 July 2017 | PR-17049 |

| LSHTM | London School of Hygiene & Tropical Medicine | 24 May 2017 | 12218 |
| --- | --- | --- | --- |

# Appendix S3 – EN-INDEPTH study population – planned sampling strategy and expected weighted mean SBR from final EN-INDEPTH survey

Each site proposed an approach to reach the maximum number of births in their HDSS within the budgetary constraints, based on site-specific information from the latest HDSS census, the level of migration, geography, likely levels of non-response (either due to non-consent or unable to locate) and other factors. For the Bandim HDSS site, the urban and rural populations differ substantially in these regards, and were therefore considered separately.

Dabat and Iganga-Mayuge have bi-annual updates and do not have a specific system of pregnancy tracking therefore all women of reproductive age (15-49 years) in the HDSS site will be included in the survey.

Matlab has reliable HDSS listings of total births, and will administer the EN-INDEPTH survey to eligible women (age 15-49) in the HDSS known to have had at least one birth outcome (stillbirth or neonatal death) in the last five years. Bandim has a detailed pregnancy tracking system and they plan to select a random sample of women residing in the HDSS known to have at least one birth outcome in the last five years. The proportion of all women with a birth outcome to be reached depending on logistical and the level of mobility and out-migration.

Bandim urban site will seek to approach all women known to have had a birth outcome in the previous five years. However, it is expected that around 30% of women in the original listings will no longer be living within the HDSS, and hence that it will not be possible to interview these women. Bandim rural site will seek to approach a random sample of 80% of women recorded in the HDSS as having a birth outcome. The 80% will be selected using simple random sampling by allocating a random number to all women with a birth in the last five years in the rural HDSS. The first 24 women in each village will be approached to participate in the survey. In Bandim rural, the EN-INDEPTH survey will take place at the same time as the HDSS rounds to reduce transportation costs. It will only be possible to visit each selected women’s residence to attempt an interview twice during the data collection period, and hence it is expected that it will not be possible to reach all the women in the study area and we will expect that we will miss approximately 30% of the respondents.

Kintampo will randomly select women with at least one birth outcome in the last five years to be approached for the survey, seeking to reach the mothers of approximately 80% of the births. Assuming that around 10% of women in the previous listings have migrated outside the HDSS and a 15% non-response rate, this approach would be expected to capture around 14,677 births (see [Table 2.2](#_Table_2.2_Characteristics) for details).

# Table 2.2 EN-INDEPTH study HDSS sites characteristics

| **Characteristics** | **Bandim urban** | **Bandim rural** | **Matlab** | **Kintampo** | **Dabat** | **IgangaMayuge** | **Overall** |
| --- | --- | --- | --- | --- | --- | --- | --- |
| **Estimated number of livebirths over the five years captured within the HDSS (based on three - five year average)** | 8,384 | 20,566 | 24,315 | 23,550 | 6,600 | 11,320 | 94,734 |
| **Estimated number of still births over the five years captured within the HDSS (based on three - five year average)** | 545 | 939 | 458 | 431 | 169 | 223 | 2,765 |
| **Estimated number of total births over the five years captured within the HDSS (based on three - five year average)** | 8,929 | 21,505 | 24,773 | 23,981 | 6,769 | 11,543 | 97,499 |
| **Expected SBR (based on three - five year average)** | 61.0 | 43.7 | 18.5 | 18.0 | 25.0 | 19.3 |  |
| **Percentage of women to be approached in the survey (assume=% of total births reached)** | 100% | 80% | 100% | 80% | 100% | 100% |  |
| **Expected level of out-migration (assumed not to be influenced by parity)** | 30% | 0% | 0% | 10% | 0% | 0% |  |
| **Percentage of all women approached and still living in HDSS (=Percentage of total births in HDSS to be captured in the survey if all women are found and consent to participate)** | 70.0% | 80.0% | 100.0% | 72.0% | 100.0% | 100.0% |  |
| **Number of total births to be captured in the survey if all women still living in HDSS respond** | 6,250 | 17,204 | 24,773 | 17,267 | 6,769 | 11,543 | 83,805 |
| **Expected number of stillbirths** | 382 | 751 | 458 | 310 | 169 | 223 | 2,293 |
| **expected proportion of women still residing in HDSS that it is not possible to interview either no consent, or failed to interview after three attempts (non-response)** | 0.15 | 0.30 | 0.15 | 0.15 | 0.15 | 0.15 |  |
| **Number of total births to be captured in the survey (accounting for non-response)** | 5313 | 12043 | 21057 | 14677 | 5754 | 9812 | 68,654 |
| **Expected number of stillbirths captured in the survey based on three-five year averages** | 324 | 526 | 389 | 264 | 144 | 190 | 1,836 |
| **Overall weighted expected SBR in pregnancy history arm of survey*** |  |  |  |  |  |  | 26.7 |

* assuming that the stillbirth rate in the birth history is equal to the three-five year average of the observed stillbirth rate in the HDSS

# Appendix S4 EN-INDEPTH study consent forms

**EVERY NEWBORN ACTION PLAN**

**Partnering with ENDING PREVENTABLE MATERNAL MORTALITY**

**EN-INDEPTH CONSENT FORM FOR INTERVIEWS WITH WOMEN**

**Title:** EN-INDEPTH: Improving Household Survey Modules for Measuring Pregnancy Outcomes

**Principal Investigator:**

**Funding Source:** Children’s Investment Fund Foundation (CIFF)

**Introduction**

Good (MORNING/AFTERNOON). My name is __ (NAME). I am part of a research team from ………………………. Health and Demographic Surveillance Site (HDSS). We are located in……………………..

You are being requested to participate in a study. This form is designed to tell you everything you need to think about before you decide to agree (consent) to be part of the study or not. It is entirely your choice. If you decide to take part, you can still change your mind later on and withdraw from the study. You can skip any questions that you do not wish to answer.

Before making your decision:

- Please carefully read this form or have it read to you
- Please ask questions about anything that is not clear

Please take your time thinking about whether you would like to participate. By verbally agreeing to participate you will not give up any legal rights. If you decide to take part, you will be asked to sign a consent form or put your thumbprint in the presence of a witness, as evidence that you agreed to take part. This consent form might contain some words that are unfamiliar to you. Please ask me to explain anything you may not understand.

**Study Overview**

The purpose of this study is to improve the ways in which stillbirths and neonatal deaths are measured during household surveys, as well as to improve the methods used to determine how many weeks pregnant a mother is and the baby’s weight at birth during these surveys. It also aims to find better ways in which the HDSS can collect data on these pregnancy outcomes, in comparison to information got from the survey, so as to identify what is missed by the different methods and why.

In addition, health facility data on gestational assessment and birth weight will be linked to the HDSS data.

**Procedures**

You are being asked to participate in this study because you have been pregnant in the past five years / are currently pregnant and are living within the area where the………….HDSS does its work.

I am a trained interviewer who will ask you some questions. The interview will take about ….. minutes / hours. I am interested in your pregnancy, how many weeks you are /were pregnant and how this was measured. I would also like to know about the baby, whether the baby was born alive or not and when, if they are still alive, how much they weighed at birth and how this was measured, where you gave birth from, and other questions that I shall ask you. In addition to this interview your health facility data will be linked to the HDSS data for possible comparisons for gestational age and birth weight assessment.

I understand that telling the story may cause painful memories, in case the baby did not live. Please let me know if the interview becomes too uncomfortable or if you would like to stop the interview. I shall respect your wishes.

**Voluntary participation and withdrawal from the study**

Your participation in this activity is voluntary, that is, you do not have to participate if you do not want to, or you may withdraw from participation at any time. There is no problem with refusing to participate in the study. It is a choice that you can make.

**Risks involved**

This study only involves interviews. There shall be no procedures of any kind done to the babies or their mothers or any of the participants. We shall not give any medicine or treatment, so we do not expect any risks. However, we will ask you to give us some of your time to participate in the interview. You may also find it stressful to talk about your experience. Sick babies or mothers in the community will be directed to one of the hospitals for treatment.

**Benefits**

Your participation in this project will not provide you with any direct rewards. However, your participation will be very useful in increasing knowledge on how the good and bad outcomes of pregnancy are measured, and what the best ways to collect this information are. These methods can be used throughout the country, so that planning for babies and mothers is based on accurate information.

**Compensation**

There is no payment for your participation in this activity.

**Privacy and confidentiality**

Your answers will be private. The research records will be kept under lock and key. A study number rather than your name will be used on study records. The records will only be accessible to the research team. On completion of the research, records will still be stored securely for the required time and later destroyed. Your name and other facts that might point to you will not appear when we present this study or publish its results.

**Contact information**

If you have any questions about this study, please feel free to ask me. You can also contact the members of the research team using the following contacts: ….…………………………………………………………

If you have questions, concerns or complaints about the research or about your rights as a participant, please contact:

IRB contact…………………………………………………………………………………….

**Consent**

Do you understand or have any questions about anything I just said? Do you agree to all of the above and to take part in the study?

If you agree to participate in the study please indicate this by signing this consent form. We will give you a copy to keep.

*I have understood what this study is about and I have agreed to participate in it. I accept that I can withdraw at any time.*

Name of Subject

Signature / thumb print of subject Date Time

*I have provided the participant with the required information about the study, and they understand the processes and what it involves*

Name of individual conducting consent discussion

Signature of Individual Conducting Consent Discussion Date Time

*NOTE: Give the participant a copy of this form. The Principal Investigator must keep the original in the study files.*

**EVERY NEWBORN ACTION PLAN**

**Partnering with ENDING PREVENTABLE MATERNAL MORTALITY**

**EN-INDEPTH CONSENT FORM FOR FGDS WITH WOMEN**

**Title:** EN-INDEPTH: Improving Household Survey Modules for Measuring Pregnancy Outcomes

**Principal Investigator:**

**Funding Source:** Children’s Investment Fund Foundation (CIFF)

**Introduction**

Good (MORNING/AFTERNOON). My name is __ (NAME). I am part of a research team from ………………………. Health and Demographic Surveillance Site (HDSS). We are located in……………………..

You are being requested to participate in a study we are conducting to assess ways in which stillbirths and neonatal deaths measurement can be improved during household surveys as well as methods for correct gestational age and birth weight assessment.

You and other community members/stakeholders have been selected as resourceful persons to participate in this study because we regard the information that you may give to be very important.

**Purpose**

The purpose of this study is to improve the ways in which stillbirths and neonatal deaths are measured during household surveys, as well as to improve the methods used to determine how many weeks pregnant a mother is and the baby’s weight at birth during these surveys. It also aims to find better ways in which the HDSS can collect data on these pregnancy outcomes, in comparison to information got from the survey, so as to identify what is missed by the different methods and why.

**Procedures**

We are conducting focus group discussions with women who participated in the recent survey where interviewers from the HDSS came to homes and only interviewed women. The focus group discussion will last around 1-2 hours, and you will be asked to share your experiences. With permission from the group, we will audio record this interview. We will not record your name during the recording. These recordings will be kept under lock and key for 5 years according to the ethical review rules. They will be transcribed, coded and analysed by study investigators. The things you say will be combined together with what other people in the group will say and will be used to design strategies to improve measurement of pregnancies and their outcomes, gestational age, and birth weight assessment.

**Voluntary participation and withdrawal from the study**

Your participation in this activity is voluntary, that is, you do not have to participate if you do not want to, or you may withdraw from participation at any time. There is no problem with refusing to participate in the study. It is a choice that you can make.

**Risks involved**

This study only involves interviews. There shall be no procedures of any kind done to the babies or their mothers or any of the participants. We shall not give any medicine or treatment, so we do not expect any risks. However, we will ask you to give us some of your time to participate in the interview. Sick babies or mothers in the community will be directed to one of the hospitals for treatment.

We understand that some of you may have ever lost a baby, and the discussions may cause painful memories. Please let us know if the discussion becomes too uncomfortable or if you would like to withdraw from it. We shall respect your wishes.

**Benefits**

Your participation in this project will not provide you with any direct rewards. However, the results from this study are important in informing future efforts to design global strategies to improve gestational age and birth weight assessment and pregnancy and measurements of their outcomes.

**Compensation**

There is no payment for your participation in this activity.

**Confidentiality**

The results of this study will strictly be kept confidential, and used only for research purposes. Your identity will be concealed as far as the law allows. Your name will not appear anywhere on the coded forms with the information. Paper and computer records will be kept under lock and key and with password protection.

**Contact information**

If you have any questions about this study, please contact...………………………………...or any of the members of the research team.

If you have questions, concerns or complaints about the research or about your rights as a participant, please contact:

IRB contact…………………………………………………………………………………….

**Consent**

Do you understand or have any questions about anything I just said? Do you agree to all of the above and to take part in the study?

If you agree to participate in the study please indicate this by signing this consent form. We will give you a copy to keep.

*I have understood what this study is about and I have agreed to participate in it. I accept that I can withdraw at any time.*

Name of Subject

Signature / thumb print of subject Date Time

*I have provided the participant with the required information about the study, and they understand the processes and what it involves*

Name of individual conducting consent discussion

Signature of Individual Conducting Consent Discussion Date Time

*NOTE: Give the participant a copy of this form. The Principal Investigator must keep the original in the study files.*

**EVERY NEWBORN ACTION PLAN**

**Partnering with ENDING PREVENTABLE MATERNAL MORTALITY**

**EN-INDEPTH CONSENT FORM FOR FGDs WITH INTERVIEWERS**

**Title:** EN-INDEPTH: Improving Household Survey Modules for Measuring Pregnancy Outcomes

**Principal Investigator:**

**Funding Source**: Children’s Investment Fund Foundation (CIFF)

**Introduction**

Good morning/Afternoon/Evening

My name is………………………………… and am part of a research team from the Health and Demographic Surveillance Site (HDSS). We are located in…………………….. You are being requested to participate in a study we are conducting to assess ways in which stillbirths and neonatal deaths measurement can be improved during household surveys as well as methods for correct gestational age and birth weight assessment.

You and other community members/stakeholders have been selected as resourceful persons to participate in this study because we regard the information that you may give to be very important

**Purpose**

The purpose of this study is to improve the ways in which stillbirths and neonatal deaths are measured during household surveys, as well as to improve the methods used to determine how many weeks pregnant a mother is and the baby’s weight at birth during these surveys. It also aims to find better ways in which the HDSS can collect data on these pregnancy outcomes, in comparison to information got from the survey, so as to identify what is missed by the different methods and why.

In addition, health facility data on gestational assessment and birth weight will be linked to the HDSS data.

**Procedures**

We will conduct focus group discussions with interviewers who have participated in the data collection of the survey and HDSS to assess their views of the barriers or enables for reporting of pregnancy and pregnancy outcomes. The focus group discussion will last around 1-2 hours, and you will be asked to share your experiences. With permission from the group, we will audio record this interview.

We will not record your name during the recording. These recordings will be kept under lock and key for 5 years according to the ethical review rules. They will be transcribed, coded and analyzed by study investigators. The things you say will be combined together with what other people in the group will say and will be used to design strategies to improve measurement of pregnancies and their outcomes, gestational age, and birth weight assessment.

**Risks/Discomforts**

The study staff will make every effort to protect your privacy and confidentiality while you are having this group discussion. You are all informed that people’s names and discussions that took place should not be shared outside of this group. We do not expect that any physical injury or other harm not mentioned here will be incurred by participation.

It is not anticipated that any physical injury or other harm not mentioned here will be incurred by participation, but if you think you have been harmed by participating in this study, you may contact the person listed within the contacts section.

**Benefits**

You will not receive any direct benefits as a result of your participation in this study. However, the results from this study are important in informing future efforts to design global strategies to improve gestational age and birth weight assessment and pregnancy and measurements of their outcomes.

**Voluntary participation and withdrawal from the study**

Your participation in this study is voluntary and you are free to take part or withdraw at any time.

**Confidentiality**

The results of this study will strictly be kept confidential, and used only for research purposes. Your identity will be concealed as far as the law allows. Your name will not appear anywhere on the coded forms with the information. Paper and computer records will be kept under lock and key and with password protection.

**Contact information**

If you have any questions about this study, please contact...………………………………...or any of the members of the research team.

If you have questions, concerns or complaints about the research or about your rights as a participant, please contact:

IRB contact…………………………………………………………………………………….

**Consent**

I confirm that the nature and the benefits of this research have been explained to me and I understand and accept them. I have also read /listened and understood this consent form, and I accept to participate in this research. The interviewer has discussed this information with me and offered to answer my questions. For any further questions, I may call ………………………………………….

Name of Subject

Signature / thumb print of subject Date Time

*I have provided the participant with the required information about the study, and they understand the processes and what it involves*

Name of individual conducting consent discussion

Signature of Individual Conducting Consent Discussion Date Time

*NOTE: Give the participant a copy of this form. The Principal Investigator must keep the original in the study files.*

# Appendix S 5 – EN-INDEPTH study questionnaire (a separate Excel file)

# Appendix S 6 – EN-INDEPTH study fieldworkers questionnaire (a separate Excel file)
